# Supplementary material for: Empirical methods for controlling false positives and estimating confidence in ChIP-Seq peaks
Source: BMC Bioinformatics. 2008 Dec 5;9:523. doi: 10.1186/1471-2105-9-523 (PMC2628906; doi:10.1186/1471-2105-9-523)
Supplement: Additional File 1 — A variety of html documents from the USeq web site detailing the available applications, their best usage, output file type descriptions, command line menus, etc. [file 1471-2105-9-523-S1.zip › USeqUserGuides/JavaDoc.zip!/JavaDoc/selex/class-use/SelexParams.html]

Uses of Class selex.SelexParams


|  |  |  |
| --- | --- | --- |
| **Overview** \| **Package** \| **Class** \| **Use** \| **Tree** \| **Deprecated** \| **Index** \| **Help** | |  |
| PREV   NEXT | **FRAMES**    **NO FRAMES**     **All Classes** |  |


---


## **Uses of Class selex.SelexParams**

| Packages that use SelexParams | |
| --- | --- |
| **selex** | For processing of raw SELEX sequence data. |

Uses of SelexParams in selex

Constructors in selex with parameters of type SelexParams

`SeqFiles(java.lang.String aSeqFile,
java.lang.String aQualFile,
SelexParams SP)`

`SeqRead(java.lang.String sequence,
java.lang.String seqComment,
java.lang.String quality,
java.lang.String qualComment,
SelexParams aSP,
SeqFiles aSF)`

`SubSeq(java.lang.String sequence,
int[] qualityScores,
SelexParams SP,
SeqRead srObjectRef)`

---


|  |  |  |
| --- | --- | --- |
| **Overview** \| **Package** \| **Class** \| **Use** \| **Tree** \| **Deprecated** \| **Index** \| **Help** | |  |
| PREV   NEXT | **FRAMES**    **NO FRAMES**     **All Classes** |  |


---
